# Primary Epithelioid Angiosarcoma of the Thyroid in a Patient Occupationally Exposed to Radiations

**DOI:** 10.3389/fendo.2018.00577

**Published:** 2018-10-01

**Authors:** Michela Marina, Luigi Corcione, Maria Francesca Serra, Teore Ferri, Enrico Maria Silini, Graziano Ceresini

**Affiliations:** ^1^Endocrinology of Aging Unit, Department of Medicine and Surgery, University of Parma, Azienda Ospedaliero-Universitaria di Parma, Parma, Italy; ^2^Pathology Unit, Department of Medicine and Surgery, University of Parma, Azienda Ospedaliero-Universitaria di Parma, Parma, Italy; ^3^Otolaryngology Unit, Department of Medicine and Surgery, University of Parma, Azienda Ospedaliero-Universitaria di Parma, Parma, Italy

**Keywords:** epithelioid, angiosarcoma, thyroid, radiation, survival

## Abstract

**Background:** Angiosarcoma (AS) of the thyroid is a rare and aggressive tumor. Its incidence is higher in iodine-deficient areas but cases unrelated to endemic goiter have been reported.

**Case Presentation:** We describe a case of a 63-year-old Italian man living in a non-iodine-deficient area, with no previous diagnosis of thyroid disease with a history of radiation exposure. The patient—an interventional cardiologist who had worked for 15 years in an angiographic room- came to the clinical observation because of the rapid onset of dyspnea and dysphonia. Computed tomography (CT) showed a 13-cm inhomogeneous neck mass, originating from the left thyroid lobe which caused displacement and stenosis of the trachea. The patient underwent diagnostic fine-needle aspiration that was followed by total thyroidectomy and lymphadenectomy of central and left lateral cervical nodes. The final pathological diagnosis was epithelioid angiosarcoma (EAS), high grade. The preoperative staging by CT of the head, neck, abdomen, chest and pelvis was negative. At pathological staging, the tumor was angionvasive but it was limited to the thyroid; no lymphnode metastases were detected. Chemotherapy with Epirubicin and Ifosfamide was administered for 4 cycles and, then, it was discontinued due to significant bone marrow toxicity.

**Conclusion:** One year after diagnosis, the CT of neck, abdomen, chest, and pelvis were negative. At 2 years after diagnosis, the FDG-PET was negative with no evidence of the disease at CT Due to the known association between the occurrence of angiosarcoma after radiation therapy it is tempting to speculate that in this patient the presence of thyroid EAS may be linked to radiation exposure.The patient is still alive at 62 months after diagnosis. He is on a follow-up program by a 6-month /1-year neck, chest, abdomen, and pelvis CT evaluation with no signs of metastases.

## Backround

Thyroid angiosarcoma (AS) is a rare, aggressive, mesenchymal tumor of the thyroid gland with vascular differentiation [1]. It mainly occurs in adult females; the highest incidence is reported in the seventh decade [2–4]. Its prognosis is considered very poor with early metastases occurring at lymphnodes, lung, skin, bones, soft tissues, and with a mean overall survival of 6 months [2, 3, 5–7]. Thyroid AS usually presents as a large and hemorrhagic thyroid mass that extends to local tissues, lymphnode, and distant sites. The non-neoplastic gland frequently shows multinodular goiter.

Thyroid AS was originally described in iodine-deficient areas of the Alps and other mountain regions in association with endemic goiter. It accounts for up to 4.3% of all malignant thyroid tumors in Switzerland [8] and its presence is documented in other mountain regions such as Austria and Northern Italy [9]. Although there have been case reports of AS in patients without goiter, many patients may not be aware of an underlying thyroid disease until a tumor is detected. Several cases have also been reported in non-alpine areas although with unknown incidence [9–14]. The coexistence of AS with Hashimoto's thyroiditis [15] or differentiated thyroid cancer [13, 16] has been reported.

Here, we report a case of primary epitelioid angiosarcoma of the thyroid diagnosed in a physician professionally exposed to radiation who lived in a non-Alpine region and had no personal history of goiter or thyroid disease.

## Case presentation

A 63-year-old man came to the clinical observation because of a rapid onset of dyspnea and dysphonia along with the development of a bulky node in the left side of the neck. He had been working as an interventional cardiologist in an angiographic room for 15 years at the local Hospital. Family history was negative with regard to malignancies and thyroid disease.

The relevant medical history included hypertension treated with valsartan and hydrochlorothiazide and non-insulin-dependent diabetes mellitus treated with metformin. There was no previous history of thyroid disease. Two years before the admission, he was treated with warfarin because of a deep venous thrombosis of the left leg occurred after a short bed rest for prostatitis. He was a heavy smoker.

The iodine status of the patient was not known; however, he was from a non-Alpine region and he was still living in the same area which is considered as a mildly iodine insufficient [17].

On physical examination, the patient had a 8 × 10-cm firm left-sided neck mass with a right-sided shift of the larynx. On ultrasound examination, a nodule of the left thyroid lobe was found measuring 5 and 6-cm in its antero-posterior (AP) and transverse (T) diameters, respectively. The nodule was hypoechoic but inhomogeneous, with no vascularization; at the strain elastography, the nodule ranged from a medium elasticity to a hard pattern. The volume of the right thyroid lobe was reduced with a small hypoechoic nodule. No enlarged lymphnodes were found at the neck ultrasound. Computed tomography (CT) of the neck confirmed a 7 × 5 × 13-cm (T × AP × Long diameters) large, inhomogeneous neck mass originating from the left lobe that caused displacement of the trachea, the left common carotid artery and the left internal jugular vein. No evidence of primary malignancies or suspicious for secondary lesions was found at the CT of the head, abdomen, and pelvis. The chest CT showed a 6-mm round-shaped nodule not suspicious for malignancy close to the parietal pleura at the lower lobe of the right lung.

**Figure 1:**
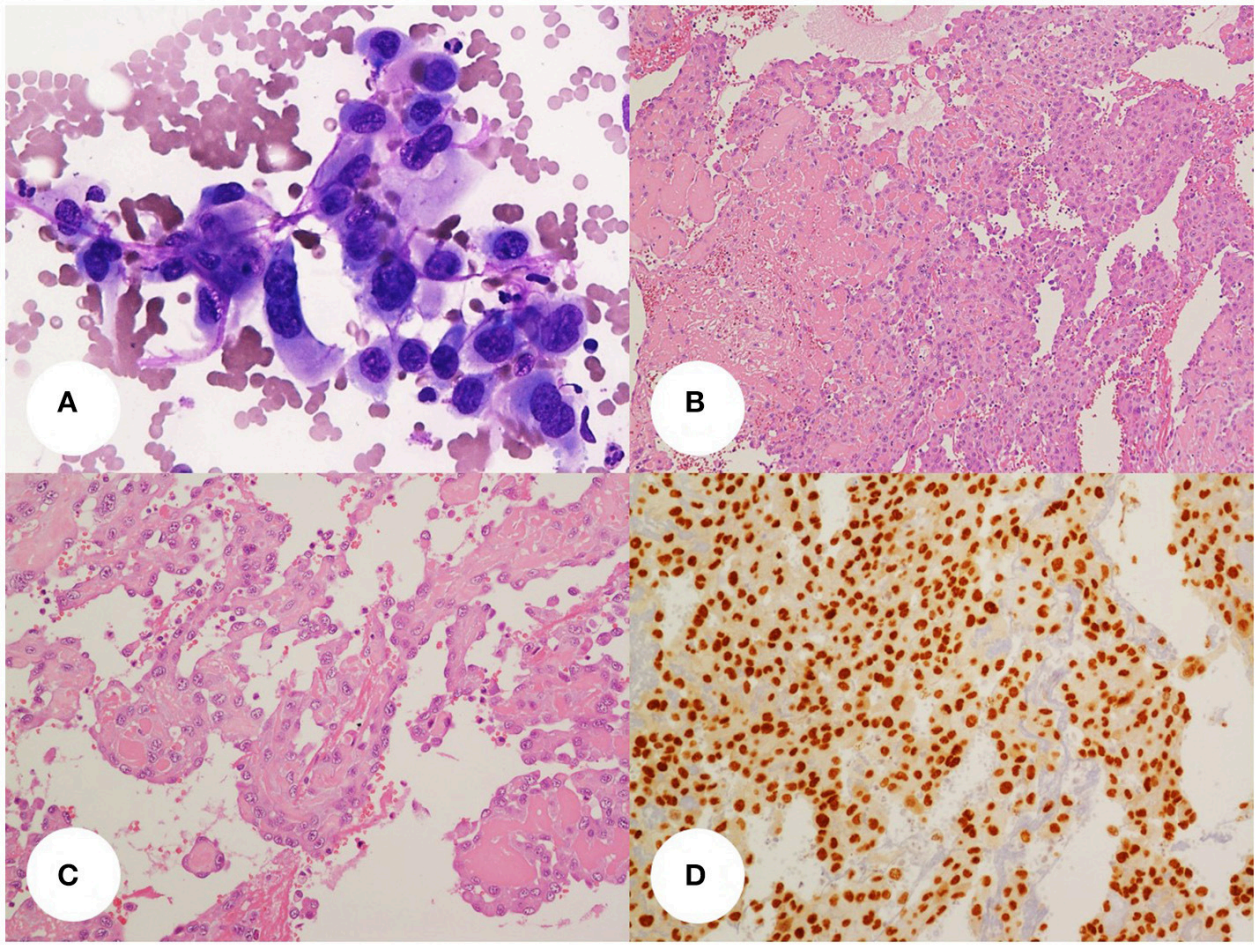
**(A)** thyroid fine-needle aspiration smear(400x, Giemsa stain) showing a small dishesive group of large, atypical, epithelioid cells with well-defined cell borders, pleomorphic and vesicular, central nuclei and eosinophilic cytoplasms. **(B,C)** Histology slides (**B**, 100x; **C**, 200x; H&E stain) show the above cells organized in sheets and papillae with fibrin cores or lining irregular vascular. Areas of recent and remote hemorrhage with siderophages can be seen. The tumor cell delimiting the vascular spaces are dishesive and pluristratified. Some cells show small intracytoplasmic lumina. **(D)** the tumor cells display diffuse nuclear stain for the vascular transcription factor Erg-1 (200x, DAB stain).

A fine needle aspiration cytology (FNAC) of the mass was performed which yielded hemorrhagic smears with few groups of large, epithelioid cells, with vesicular, severely atypical nuclei and eosinophilic dense cytoplasms. A diagnosis of malignancy was given (Category 6 according to Bethesda 2010) with a suggestion for an anaplastic carcinoma (Figure 1A).

The patient underwent a total thyroidectomy and lymphadenectomy of central and left lateral cervical nodes. At the gross pathology examination, the tumor measured 6 × 6 × 12 cm (T × AP × Long diameters) and was partially circumscribed by a fibrous pseudocapsule. The mass had a gray, tan and red cut surface, with areas of hemorrhagic necrosis. Histology showed a vasoformative high grade neoplasia characterized by large epithelioid cells growing in sheets and lining abnormal vascular spaces; some cells showed intracytoplasmic lumina. There were areas of spontaneous necrosis and hemorrhage and a brisk mitotic activity; angioinvasion was noted. The tumor immunostained for vascular markers (CD31, ERG, CD34, factor VIII and vimentin), whereas epithelial differentiation markers were negative (cytokeratins, thyroid transcription factor 1, thyroglobulin, and EMA). The final histologic diagnosis was primary epithelioid angiosarcoma of the thyroid, grade 3 according to FFCCS (Figures 1B–D). This diagnosis was confirmed at a second opinion from a different institution. The tumor was restricted to the thyroid with free surgical margins. The mass had substernal extension and displaced the surrounding structures but it did not infiltrate the thyroid capsule, the strap muscles, or other neck tissues. The remaining thyroid tissue had nodular colloid goiter. No lymphnode metastases were detected.

Fifteen days after the thyroidectomy, the patient was operated to prevent rupture of an aneurysm of the abdominal aorta. One month after thyroidectomy, the chest CT showed multiple pulmonary nodular lesions some of them with a solid pattern surrounded by a ground-glass halo, 12 mm in maximum diameter. There was no consensus as to the oncologic relevance of these lesions, therefore, no biopsy was performed. A bone scintigraphy yielded negative results.

Chemotherapy with Epirubicin, Ifosfamide, and Mesna was administered but it was discontinued after 4 cycles because of pancytopenia and infection by *Klebsiella Pneumoniae*, treated with piperacillin/tazobactam, and by *Clostridium difficile*, treated with vancomycin. The patient recovered from the infections and, at a 6-month follow-up, the chest CT showed a reduction of the number and volume of the lung lesions with only three of them remaining in the medial lobe of the right lung.

At a further 18-month control, the chest CT was unchanged. The 6 mm round-shaped nodule close to the parietal pleura at the lower lobe of the right lung was also found to be stable. One year later, the patient developed pneumonitis and recovered after antibiotic therapy. At that time, he was investigated by neck, chest, abdomen and pelvis CT as well as with FDG-PET without any evidence of disease recurrence.

Afterward, a 6-month CT follow-up program was started which is still ongoing. At present, the patient is alive with no evidence of disease after 62 months from initial diagnosis.

## Discussion

A recent systematic review [18] indicates that the majority of thyroid AS cases are still diagnosed in people living in alpine regions of Europe, especially Switzerland, Northern Italy and Austria and they are associated with endemic nodular goiter [1, 8, 19, 20]. The association between AS and nodular goiter, however, is not absolute and up to 50% of thyroid AS may arise in different contexts [3, 12, 14, 21].

In the liver, AS has been observed in subjects exposed to vinyl chloride, arsenic, and thorium dioxide (Thorotrast) [1]. The occurrence of AS after radiation therapy is also well documented [22, 23], although mainly for superficial rather than visceral sites [1, 22, 24]. It has been hypothesized that this association between radiation therapy and the development of AS can be due to stasis within lymphatic channels due to the development of fibrosis or a radiation-induced sarcoma [25–27]. Potential risk factors, apart from long-standing goiter, have been rarely recognized and reported in thyroid AS cases. In 2016, Collini et al. in a series of six cases of thyroid AS from a non-iodine-deficinent area found a male patient with occupational exposure to vinyl chloride and a female patient with a history of radiation therapy for malignant timoma [22]. No known risk factor was recognized for the other patients. The patient herein reported had worked for 15 years in an angiographic room as an interventional cardiologist. Although no data are available as to the radiation dose to the thyroid, it is tempting to speculated a link between this professional exposure and the development of AS that should alert for the occurrence of similar cases.

Histologically, the morphology of thyroid AS may range from well-differentiated forms, to poorly differentiated tumors with solid growth of spindle and/or epithelioid cells. The main differential diagnosis is with anaplastic thyroid carcinoma or other high-grade sarcomas. The diagnosis is now made easy by the availability of several antigenic markers; this does not necessarily apply to old literature data.

The patient had a R0 thyroidectomy with no extra-thyroidal invasion or lymphnode disease and, despite angionvasion and an incomplete chemotherapy treatment due to infectious complications, he remains free of disease at 62 months from diagnosis. The reported prognosis of patients with thyroid AS is poor and survival rates are limited to few months after the initial diagnosis [2, 3]. However, single cases with longer survival (in one case, up to 82 months) have been reported [22].

Angionvasion has been suggested as a possible risk factor for progression on a limited series of cases and review of the literature [28]. In larger series, tumors confined to the thyroid and without distant metastases at diagnosis seem to have a better prognosis [18, 22, 29].

There is no established therapeutic strategy for post-surgical management of thyroid AS. Some authors reported promising results with adjuvant radiotherapy [9, 30–32], although its role still remains unclear. Also the role of chemotherapy remains to be elucidated. Several drugs have been used, such as epirubucine, adriamicine, taxanes, and ifosfamide, either alone or in combination. Chemotherapy has been used either in adjuvant or neo-adjuvant protocols as well as in combination with radiotherapy [18] but the overall outcome is poor. New treatment strategies are under investigation, including drugs targeting the vascular endothelial growth factor (VEGF) and its receptor (VEGFR) pathway (i.e., bevacizumab) as well as tyrosine kinase inhibitors with activity against VEGFR (i.e., sunitinib and pazopanib) [33, 34]. Also, inhibition of phosphatidylinositol 3-kinase pathway has been hypothesized to virtually represent a further therapeutic tool, based on the findings of the association between this pathway and AS, although of non-thyroidal origin [35, 36]. However, further studies are needed in order to verify and confirm the effects of these novel therapeutic approaches on thyroid AS.

Our patient was treated with epirubicin in combination with ifosfamide. The therapy was discontinued after four cycles of the 6-cycles planned treatment because of severe bone marrow toxicity. Nevertheless, no evidence of disease is still recorded at the follow-up. Notably, Maiorana et al. reported a thyroid AS with 66-month disease-free survival in a patient with no treatments after surgery [11]. Undoubtedly, the critical issue in discussing the clinical course is the limited clinical experience available for this tumor and the related difficulty to properly design therapeutic protocols.

No evidence-based explanation can be provided for the favorable clinical course so far observed in our patient. The expansile pattern of growth of the tumor that was limited to the thyroid and lacked distant metastases are pathological variables that suggest a less aggressive behavior [37]. Angioinvasion is the norm in vaso-formative tumors and seems of little consequence in this setting. Conversely, it is tempting to speculate that AS arising in different settings than goiter may have a lower biological potential than tumors complicating endemic iodine deficiency which still make most of the current literature on this subject. More attention should be given to this issue in future studies.

## Ethics statement

The patient gave written informed consent for the publication of this report.

## Author contributions

All authors listed have made a substantial, direct and intellectual contribution to the work, and approved it for publication.

### Conflict of interest statement

The authors declare that the research was conducted in the absence of any commercial or financial relationships that could be construed as a potential conflict of interest.
